# Human Papilloma Virus (HPV) driven oropharyngeal cancer in current or previous heavy smokers: should we look for a different treatment paradigm?

**DOI:** 10.3389/fonc.2024.1383019

**Published:** 2024-04-08

**Authors:** Luigi Lorini, Paolo Bossi, Amanda Psyrri, Pierluigi Bonomo

**Affiliations:** ^1^ Medical Oncology and Hematology Unit, IRCCS Humanitas Research Hospital, Rozzano (Milan), Italy; ^2^ Department of Biomedical Sciences, Humanitas University, Pieve Emanuele, Milan, Italy; ^3^ Section of Medical Oncology, 2(nd) Department of Internal Medicine, School of Medicine, National and Kapodistrian University of Athens, Attikon University Hospital, Haidari, Athens, Greece; ^4^ Radiation Oncology Unit, Azienda Ospedaliero-Universitaria Careggi, Florence, Italy

**Keywords:** HPV/related oropharyngeal squamous cell carcinoma, smoking, radiation, treatment escalation, locally advanced head and neck squamous cell carcinoma

## Abstract

**Introduction:**

Human papillomavirus Virus (HPV)-associated oropharyngeal squamous cell carcinoma (OSCC) has increased in incidence in recent decades and represents a heterogeneous disease entity in the context of Head and Neck Squamous Cell Carcinoma (HNSCC), in terms of disease prognosis. Treatment of locoregionally advanced OSCC is mainly based on concurrent chemoradiotherapy. Given the younger age of patients, if compared with HPV-negative counterparts, and the high cure rates, the acute- and long-term toxicity in survivors represents a field of interest. However, patient selection for de-escalation trials remains a major challenge due to the lack of robust validated prognostic indicators within the HPV-associated OSCC.

**Discussion:**

The impact of smoking status on HPV-associated OSCC prognosis has been demonstrated in the majority of studies. However, the magnitude of the association is unclear due to variability in smoking metrics and study outcomes. Smoking status has been identified as a potential confounding factor in HPV-positive de-escalation trials. Smokers with HPV-positive OSCC have a worse prognosis in most studies than non-smokers and may require different and more aggressive therapeutic strategies.

## Introduction

### Human papilloma virus (HPV) and oropharyngeal squamous cell carcinoma (OSCC): is there still anything to delve into?

The recognition of Human Papilloma Virus (HPV) as a main pathogenetic factor for oropharyngeal squamous cell carcinoma (OSCC) revolutionized the approach to head and neck cancer: the long-term follow-up of the pivotal RTOG 0129 trial (1, 2) confirmed the existence of a marked difference with respect to 8-year locoregional failure and overall survival between HPV positive and negative patients (19.5% vs 52.4%, HR: 0.29, 95% CI: 0.17-0.48; 70.9% vs 30.2%, HR: 0.30, 95% CI: 0.21-0.42, p<0.01, respectively). HPV positivity has emerged as an independent favorable prognostic both in progression free survival and overall survival (OS), leading to the introduction of p16 status in the AJCC TNM 8^th^ version (3). HPV-driven OSCC is considered a radiosensitive disease, in the majority of cases, and this radiosensitivity is much likely driven by inactivated – but not mutated – p53 response pathway mediated by the activity of HPV E6-oncoprotein (4–6).

However, not all HPV positive patients fare well after standard treatment. HPV-mediated carcinogenesis relies on the integration of the viral DNA into the host genome, loss of HPV early gene 2 (HPV E2) expression, the transcriptional repressor of viral E6/E7 gene expression, activation of Phosphatidylinositol-4,5-Bisphosphate 3-Kinase Catalytic Subunit Alpha (PIK3CA), and apolipoprotein B mRNA editing catalytic polypeptide (APOBEC)-mediated mutagenesis. Schrank TP et al. (7) by analyzing transcriptional data from 104 HPV-driven head and neck squamous cell carcinoma (HNSCC) together with two publicly available sources were able to identify a subclass of HPV+ carcinomas comprising ~45% of HPV-driven HNSCC that is not associated with any of these typical features. The subclass-defining gene set was strongly correlated with Nuclear factor kappa B (NF-κB) target expression and harbored inactivating alterations of key regulators of NF-κB, TNF receptor associated factor 3 (TRAF3), and cylindromatosis (CYLD), as well as retinoblastoma protein (RB1). Cellular model with experimental inactivation of these NF-κB regulators demonstrated increased sensitivity to radiation.

Other molecular studies also report findings (8) suggesting that about a third of patients present PIK3CA mutations or amplifications, which may be associated with worse outcome, and three molecular clusters, based on transcriptomic data, may be identified, with different prognoses, namely immune-related; epithelial-mesenchymal related; and proliferation-related; an immune-based classification based on transcriptomic features showed a strong correlation with outcome (9).

### Smoking behavior and HPV status

Smoking status may mitigate the effect of HPV positivity in OSCC, acting as a potential proxy for a dual etiopathogenesis of the disease. Back to 2010, in the RTOG 0129 trial (1), a recursive partitioning analysis showed that the number of pack/years of smoking (¾10 vs >10) was second only to HPV status as a determinant of OS. Thus, they divided OSCC population into three groups based on risk factors: low and high risk groups were represented by HPV positive and negative patients, respectively; intermediate risk patients (representing 29.7% of the patients) comprised both HPV positive with smoking history > 10 pack/years and/or a high nodal stage (N2b-N3) and HPV negative, stage T2 and T3 and with low smoking history (¾ 10 pack/years). The 3-year OS rate of low, intermediate, and high risk OSCC was respectively 93%, 70%, and 46%, thus confirming different prognosis among HPV positive tumors. The increased risk of death has been quantified as 1% per pack year and 2% per year of smoking (10). Data have been confirmed in a large dataset of 5 randomized trials (RTOG 9003 and 0129, DAHANCA 6&7 and ARTSCAN) (1, 11–14), where in p16+ tumors never smokers had significantly better PFS than former/current smokers (HR = 0.49 [0.33; 0.75]), with survival benefits at 3, 5 and 10 years of +20%, +18.7%, and +24.2% respectively. Two further relevant aspects make the interpretation of these clinical findings even more complicated. First, the pivotal HNCIG-EPIC-OPC multinational individual data analysis on 7654 patients suggested that the identification of HPV-positive status should rely on HPV testing and not only on p16 assessment (as indicated in the AJCC8 staging system), in view of the worse prognosis showed in case of discordant results (15). Second, the modulating effect of smoking on treatment efficacy is an object of controversy due to the lack of clear thresholds. In fact, both the 10 and 20-pack year cutoffs have been employed in different trials, as well as the current or former smoking status.

Biological basis explaining different outcomes within HPV-driven OSCC smokers is not well understood. Changes in immune microenvironment have been shown among smoking HPV OSCC patients, with a lower level of immune infiltration, lower cytolytic activity, and lower IFN gamma pathway signaling (16). Wahle B et al. (17) showed that the low-risk group is associated with high expression of T cell-related transcripts. OP-TIL, a biomarker that characterizes the spatial interplay between tumor-infiltrating lymphocytes (TILs) and surrounding cells in histology images, separated stage I HPV-associated OSCC patients with 30 or less pack-year smoking history into low-risk and high-risk groups. Moreover, conflicting data exist on differences in mutational profile between HPV positive OSCC smokers and no smokers. One series showed higher TP53, CDKN2A, KRAS, and NOTCH1, and lower HLA-A mutations in smokers (14), while another one showed no differences (18). Villepelet et al. (19) showed that chromosomal aberrations and breakpoints are more frequent in HPV+ smokers. To the contrary, dose-dependent increase in the smoking-related signature strength with greater smoking exposure was reported by Schrank T et al. (20). Beyond mutations, tobacco also induces epigenetic alterations. These molecular deregulations, coupled with the reduction of immune inflammatory pathways and the increase in tumor hypoxia, may create a tumor environment more resistant to radiation therapy than what happens in “pure” HPV-related, non-smoking cancer patients.

## Discussion

### New approaches for HPV-driven OSCC with smoking history: is the evidence yet inconclusive?

De-escalation strategy to maintain treatment efficacy with lower toxicity in HPV-driven patients has been the object of several therapeutic strategies so far. However, the HPV-positive smoker group may deserve further specific data, based on the hypothesis that de-escalation could compromise survival in such patients given that specific data on such population are lacking. In the RTOG 0129 no specific outcomes were reported for the subgroup of HPV-positive patients included in the intermediate risk group. Such lack of distinction between low and intermediate risk group may impair trial results, as for the phase III RTOG 1016 trial (21), designed with de intensification purpose, in which most patients with HPV-positive disease could be enrolled and smoking status appeared only as stratification (threshold: 10 pack years) and not as a selection factor. Even if not powered for subgroup analysis, the 5-year OS for the intermediate group was 76.4% and 71.4% for the cisplatin and cetuximab arm, respectively. On the other side, even intensification trials did not separately consider intermediate risk and high risk OSCC, with the consequent risk to underestimate the benefit of treatment for the intermediate risk group. One such example is the multi-arm, controlled randomized phase 3 COMPARE trial (Comparing Alternative Regimens for escalating treatment of intermediate and high-risk oropharyngeal cancer). Based on the first results of the trial presented at ICHNO meeting in 2022 by Mehanna et al, dose-escalated chemoradiation (64 Gy in 25 fractions with concomitant cisplatin) was not associated with any benefit compared with standard treatment in 257 patients with oropharyngeal cancer, of whom 80% had an intermediate risk (22). At ESMO 2023 Zandberg et al. presented the updated results of a randomized, phase 2 study evaluating concurrent or sequential fixed-dose pembrolizumab in combination with cisplatin-RT. Either intermediate or high risk OSCC patients were enrolled. Sequential pembrolizumab had a numerically superior 1 and 2-year PFS compared to concurrent therapy with lower toxicity burden. However specific data for intermediate risk group are not available (23).

However, the worsening effect of smoking in HPV-positive cancer might depend on the primary therapeutic strategy. In the largest, transoral-surgery based randomized phase II trial published to date – the ECOG 3311 trial the intermediate risk HPV positive patients (n=182), current or former smokers (>10 pk/years) had no worse outcome than those with a mild lifelong tobacco exposure (<10 pk/years) (24). Main findings of cited escalation/de-escalation treatment trials are summarized in **Table 1**.

**Table 1:** Prospective study on escalation/de-escalation treatment for LA HNSCC and LA OSCC.

First author	Year of pubblication	Study Population	Number of Patients	Type of treatment	Primary Endpoint	Primary Endpoint Results
**Gillison** (21)	2019	OSCC P16 positive T1-T2, N2a-N3 or T3-T4, N0- N3 M0 (AJCC VII edition)	805	RT (70 Gy) + cisplatin vs RT (70 Gy) + cetuximab	5-year OS	84,6% (cisplatin arm) vs 77.9% (cetuximab arm)
**Sanghera** (22)	2022	OSCC p16 positive or negative If p16 positive: N2b or above disease and tobacco smoking history ‗ 10 pack/years	257	Different arms. ARM 1: RT 70 Gy + cisplatin ARM 3: RT 64 Gy + cisplatin	EFS and OS	3-year EFS:72% (ARM 1) v 68% (arm 3) 3-year OS: 79% (ARM 1) vs 74% (arm 3)
**Zandberg** (23)	2022	LA HNSCC (p16 positive and p16 negative) If OSCC p16 positive: T4 or N3 or tobacco smoking history ‗ 10 pack/years	80	RT (70 Gy) + cisplatin + concomitant pembrolizumab vs RT (70 Gy) + cisplatin + sequential pembrolizumab	1-year PFS	89% (sequential arm) vs 82% (concomitant arm)
**Tao** (25)	2023	LA HNSCC (stage III, IVa, and IVb, limited to T ≥2, N0–3, and M0 (AJCC VII edition); tobacco smoking history of ‗10 pack/years	96	RT (70 Gy) + cisplatin + xevinapatin vs RT (70 Gy) + cisplatin + placebo	LRC at 18 months	54% (xevinapant arm) vs 33% (placebo arm)

LA HNSCC (locally advanced head and neck squamous cell carcinoma); OSCC (oropharyngeal squamous cell carcinoma); RT (radiotherapy); Gy (Gray); AJCC (American Joint Committee on Cancer); EFS (event free survival); OS (overall survival); LRC (locoregional control).

A specific focus on intermediate risk group has been made in the ongoing randomized phase 2 CCTG HN9- EORTC 1740 trial (NCT03410615), which evaluates different combinations of durvalumab and tremelimumab with radiation over concomitant chemoradiation alone; patients with T1-2N1M0 or T3N0-N1 with a smoking history > 10 pack/years could be enrolled; trial is closed but data have not been presented, yet.

### Should we look for a different treatment paradigm in HPV positive patients with significant tobacco exposure?

The challenge in intermediate risk is to safely intensify the treatment. This group is heterogeneous, including both smokers with high nodal burden HPV-positive status and non-smokers with T2 and T3 HPV-negative status according to Ang’s classification. It is important to consider that the grouping in question is more complicated because it doesn’t include the T4 stage as a worsening factor in HPV-positive cases. It’s uncertain whether this stage should be included in the definition of the intermediate group or not, and further clarification is needed. While the number of de-escalation trials in low-risk HPV-positive cancers is increasing, there is no similar trend for escalating treatment in worse-prognosis HPV-positive patients.

Trials should target the patients with HPV-positive cancer and added risk factors, namely smoking, higher tumor stage, and molecular features, in a way to increase survival without impacting toxicities.

Xevinapant, a first-in-class, potent, oral, small-molecule, targeting inhibitor of apoptosis protein (IAP) is emerging as a promising compound. Extended follow up data of the phase 2 trial recently published confirms xevinapant’s activity, efficacy, and safety profile in high risk locally advanced HPV negative head and neck squamous cell carcinoma combined with standard cisplatin based RT (25). Due to its modality of action, it is reasonable that the addition of this agent could exert its activity also in HPV-positive cancers with an history of smoking. However, so far, only 3 out of 48 patients allocated in the experimental arm of the aforementioned randomized phase 2 trial were HPV positive (25), thus no clinical evidence supports the biologic assumption of activity in this patient population, for the time being.

## Conclusion

In conclusion, there is an unmet need to identify an alternative treatment paradigm for intermediate risk HPV-positive OSCC, that could consider the lower prognosis of this subgroup of patients, when compared to the low-risk group of HPV-positive cancer patients without a smoking history, and that would not impact too much in treatment-induced toxicities.

## Data availability statement

The original contributions presented in the study are included in the article/supplementary material, Further inquiries can be directed to the corresponding author.

## Author contributions

LL: Conceptualization, Validation, Writing – original draft, Writing – review & editing. PoB: Conceptualization, Supervision, Validation, Writing – original draft, Writing – review & editing. AP: Writing – original draft, Writing – review & editing, Conceptualization, Validation. PiB: Conceptualization, Data curation, Investigation, Methodology, Writing – original draft, Writing – review & editing.
